# Laser Spot Micro-Welding of Ultra-Thin Steel Sheet

**DOI:** 10.3390/mi12030342

**Published:** 2021-03-23

**Authors:** Quanhong Li, Zhongyan Mu, Manlelan Luo, Anguo Huang, Shengyong Pang

**Affiliations:** State Key Laboratory of Materials Processing and Die & Mould Technology, School of Materials Science and Engineering, Huazhong University of Science and Technology, Wuhan 430074, China; m201870963@hust.edu.cn (Q.L.); muzy@hust.edu.cn (Z.M.); luomanlelan@hust.edu.cn (M.L.); huang-anguo@hust.edu.cn (A.H.)

**Keywords:** micro-welding process, laser pulse waveform, coupling optimization, ultra-thin plates, numerical simulation

## Abstract

This paper reports a mechanism understanding how to reduce the solder joint failure phenomenon in the laser spot micro-welding process of ultra-thin steel sheets. An optimization method to improve solder joint service life is proposed. In this study, the time-dependent dynamic behaviors of the keyhole and the weld pool are simulated, and the temperatures in the keyhole of two different laser pulse waveforms are compared. The results show that laser energy attenuation mode (LEAM) can only obtain shallow weld depth because of the premature decay of the laser power of waveform, resulting in the laser beam that cannot be concentrated in the keyhole. The temperature inside the keyhole of LEAM fluctuates significantly, which shows a downward trend. Due to the existence of the peak power of waveform in laser energy continuous mode (LECM), the large angle of inclination of the wall of the keyhole inside the melt pool is more conducive to the multiple reflections of the laser beam in the keyhole and increases the absorption rate of the laser energy by the base material, resulting in the “keyhole effect”. But the temperature in the keyhole gradually rises, close to the evaporation temperature. A method combining LEAM and LECM to improve the solder joint service life by optimizing the temperature in the keyhole indirectly by adjusting the peak power of the laser pulse waveform is proposed in this study. The experimental results show that the weld depth can be optimized from 0.135 mm to 0.291 mm, and the tensile strength can be optimized from 88 MPa to 288 MPa. The bonding performance between the upper and lower plates is effectively improved. It can reach the required weld depth in a short time and improve the welding efficiency of the laser spot micro-welding process. The simulation results show that the temperature inside the keyhole is well optimized below the evaporation temperature of the material, which can avoid the violent evaporation of the welding process and keep the whole welding process in a stable state. By optimizing the laser pulse waveform, the temperature inside the keyhole can reach 3300 K, and it is always in a stable state than before optimization. The stable temperature inside the keyhole can help to reduce violent oscillation and spattering of the molten pool and improve welding efficiency and joint life. The research can help provide effective process guidance for the optimization of different laser pulse waveforms in the micro-welding process.

## 1. Introduction

Laser spot welding is an advanced micro-welding technology, which is widely used for connecting the ultra-thin plates in, e.g., aerospace, automotive, electronic products [1,2,3]. It has outstanding advantages of fast welding speed [4,5,6], high welding precision [7,8], high energy density [9,10], and environmental protection [11]. However, in the actual welding process of the manufacturing of microelectronic components, a slight splash on the surface of the parts will cause the whole component to be scrapped. Besides, there will be as many as hundreds of solder joints in a large-scale integrated circuit at the same time. If one of these solder joints fails, the whole component or the whole machine will stop working. The solder joints failure phenomenon accounts for up to 60% of all the causes of the failure of electronic components [12,13,14]. Therefore, the modern ultra-thin plates connection forming industry urgently needs to further study and optimize the micro-welding process to improve the product qualification rate and service life.

When welding ultra-thin metal materials, it is necessary to carefully adjust the heat input during the welding process to avoid welding perforation, spatter, or weak weld defects [15,16,17,18]. An in-depth exploration of the laser energy transmission mechanism in the laser spot micro-welding (LSMW) process has aroused the attention of researchers. The study of Saldi et al. [19] showed that the main process parameters affecting the laser energy were the laser pulse period. The laser pulse waveform will take on different shapes with the change in laser power and pulse period. The laser energy in the LSMW process of ultra-thin plates can be well controlled by reasonably adjusting the laser pulse waveform [20,21,22,23,24]. The reasonable application of laser pulse waveform can also reduce the common defects in the LSMW process. For example, the study of Seiji et al. [25] indicated that the porosity in the LSMW process could be effectively suppressed by cooperating laser pulse square waveform with sawtooth waveform. Besides, the cooling speed during the metal solidification process is suppressed by changing the laser pulse waveform, and the internal stress is reduced [26,27,28]. It shows that the laser pulse waveform has a great influence on the LSMW process. In recent years, more and more experimental results show that poor adjustment of laser pulse waveform will cause the metal liquid on the surface of the ultra-thin plate to oscillate violently and produce spatters [29,30,31]. Some of the available studies show that between 3.5 KW and 5.9 KW, the weld joint diameter tends to increase with increasing laser power. However, when the laser power exceeds 5.9 KW, there is a decrease in weld joint diameter. Therefore, a higher laser energy input does not necessarily lead to good welding results [32]. Pulsed laser welding is a time modulation of the laser within each pulse. When the weak weld phenomenon of solder joint occurs, or when the welding process is violently spattered, it is more inconvenient to adjust the welding parameters directly multiple times. A certain amount of experience is required. Because the laser spot micro-welding process occurs within a few milliseconds, it is difficult to directly analyze data, such as melt pool flow rate, spatter jet speed, or weld depth. At present, the research on the physical mechanism of the micro-welding process on the ultra-thin plate is lagging behind the actual application—especially in the aspects of the influence of different laser pulse waveforms on the solder joints size—and the explanation of the defect formation is relatively weak. As a result, the understanding of laser pulse waveform process optimization methods used to improve solder joint quality in practical engineering is very limited.

This paper mainly studies the effects of different laser pulse waveforms on the LSMW process in a practical welding production, and a 3D transient mathematical model coupling weld pool, laser, and the keyhole is developed to observe the LSMW process and to explain the main causes of the welding defects from the perspective of microphysics. The experimental results show that when the laser pulse energy is kept constant, the change in laser pulse duration has an obvious influence on the weld size and quality, which is caused by the change in peak power density. By processing the simulation results, we analyze the change curve of the average temperature in the keyhole during the simulation and judge whether the laser spot micro-welding process is stable under the conditions of specific process parameters according to the curve. After obtaining the more ideal welding simulation results and experimental verification through experiments and simulation, it is found that the unstable oscillation of the temperature in the keyhole is the main reason for the formation of welding defects. Finally, a method of laser pulse waveform segmented optimization is proposed, which can effectively optimize the internal temperature of the keyhole and obtain the ideal solder joints, greatly improving the qualification rate of welding components. The results can help provide effective process guidance for optimization of the laser pulse waveform in the LSMW process.

## 2. Materials and Methods

### 2.1. Experiments

**Figure 1:**
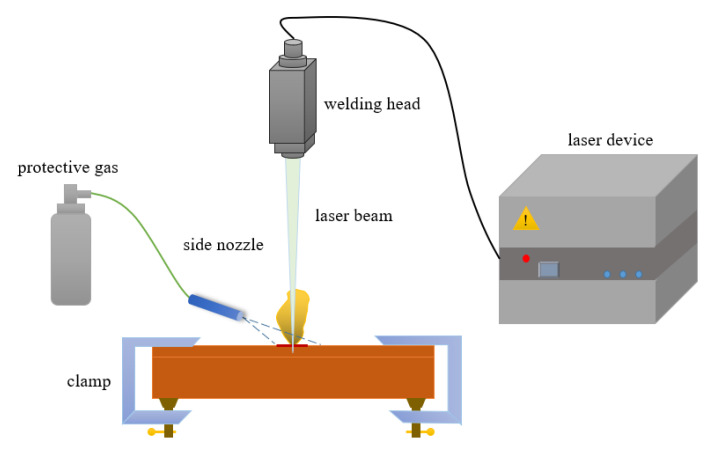
Schematic diagram of laser spot micro-welding (LSMW) experiments.

**Figure 2:**
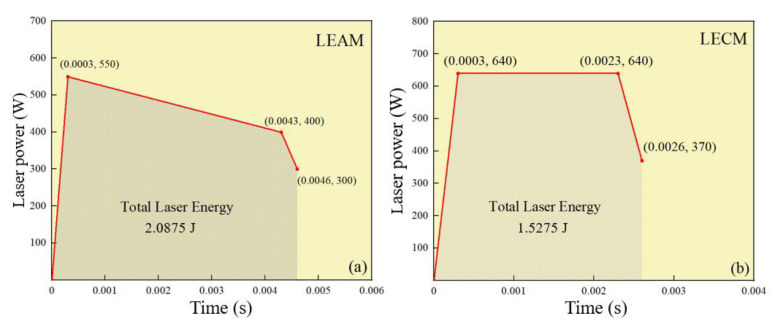
Different pulse waveforms. (**a**) Laser energy attenuation mode (LEAM). (**b**) Laser energy continuous mode (LECM).

The experiments are performed on the laser welding machine in AAC Technology, Shenzhen, China. The overall structure diagram of the experimental setup is shown in Figure 1. A pulse laser is used for welding. The radius of the laser spot at the focal position is 0.48 mm. Besides, two ANSI 304 stainless steel ultra-thin plates with a size of 0.12 mm and 0.3 mm in the penetration directions, respectively, are lapped. The chemical composition of the two plates is shown in Table 1. In welding, to study the influence of different laser pulse waveforms on the forming process of LSMW, there is mainly a variation in the peak laser power and duration. The two laser pulse waveforms are shown in Figure 2a,b. Figure 2a is laser energy attenuation mode (LEAM), and Figure 2b is laser energy continuous mode (LECM). By calculating the total energy of the laser pulse waveform, the laser input energy of LEAM is 2.0875 J, and the laser input energy of LECM is 1.5275 J. The specific experimental process parameters are shown in Table 2. Besides, argon gas is used as a welding protective gas in experiments. After the welding is done, the welds are cut with the method of wire electrical discharge machining, which shows that the effect on tensile strength is lower than 5% [33]. After that, the sample cutting is etched (Kroll reagent: 3–9 mL HCL + 1–3 mL HNO3, time 6–10 s).

### 2.2. Numerical Simulation

**Figure 3:**
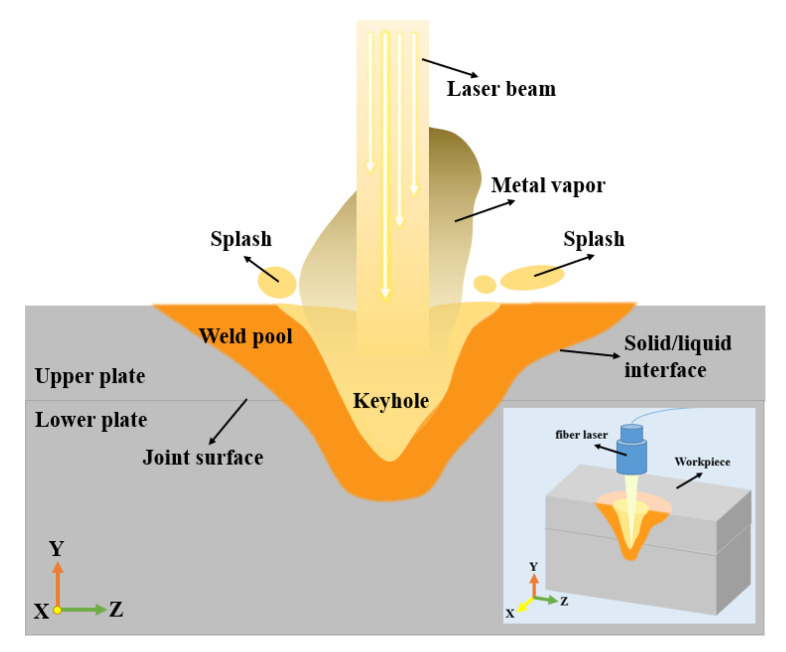
Schematic diagram of the LSMW model.

The process of laser spot micro-welding is very complex and variable due to the interaction of physical and chemical factors [34]. In this paper, a 3D mathematical model is proposed to understand the internal evolution mechanism of the LSMW process, which is shown in Figure 3. In this model, three forces of surface tension, recoil pressure, and Marangoni forces are comprehensively considered. Besides, the convection and conduction of heat and the solid-to-liquid interaction force inside the weld pool are considered. The mass, momentum, and energy conservation equations of the weld pool can be written as Equations (1)–(3) [35,36,37,38,39,40,41,42,43,44]:

(1) $$\nabla \cdot \vec{U}=0.$$

(2) $$\rho \left(\frac{\partial \vec{U}}{\partial t}+\left(\vec{U}\cdot \nabla \right)\vec{U}\right)=\nabla \cdot \left(\mu \nabla \vec{U}\right)-\nabla p-\frac{\mu }{K}\vec{U}-\frac{C\rho }{\sqrt{K}}\left|\vec{U}\right|\vec{U}+\rho \vec{g}\beta \left(T-T_{ref}\right)$$

(3) $$\rho C_{p}\left(\frac{\partial T}{\partial t}+\left(\vec{U}\cdot \nabla \right)T\right)=\nabla \cdot \left(k\nabla T\right)$$

where $\vec{U}$ means the three-dimensional velocity, p  means the pressure. ρ, μ, β mean the density, kinematic viscosity of the fluid, and expansion coefficient of the thermal, respectively. $T_{ref}$ means the reference temperature. K  means the Carman–Kozeny constant in the mixture-fluid phase model, and C means an inertial coefficient closely related to a liquid fraction. $\;C_{p}$ stands for the specific heat capacity, k stands for the thermal conductivity of the metal fluid, and T stands for the temperature.

**Figure 4:**
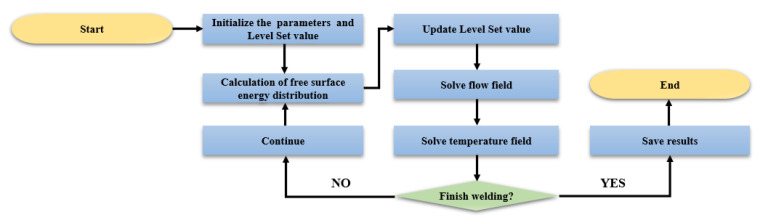
The simulation flow chart of LSMW.

Due to the extremely short welding time of the LSMW process, the level-set method is used to track the evolution process of the weld pool free interface. The laser heat source adopts the ray tracing method to consider the influence of the keyhole topography on the laser absorption rate. Based on the previous work [44,45,46,47], assuming that the energy of the laser beam is Gaussian distribution, the distribution function of laser beam energy at time t and the laser energy power P(t)  absorbed by the liquid surface micro-element at time t can be written as follows:

(4) $$Q\left(t,r,z\right)=\frac{3P\left(t\right)}{\pi R^{2}}exp\left(-\frac{3r^{2}}{R^{2}}\right)$$

where R represents the laser spot radius. For simplicity, the metal vapor and chemical reactions are not considered, and the weld pool is considered to be incompressible laminar flow in this model. Technically speaking, the precise discretization of the equations and the development of effective solvers are very important for the simulation of the LSMW process, and the specific details of the equations and parameters in the model are listed in the Appendix A. Finally, the numerical calculation and solution process of moving weld pool and transient keyhole in the process of LSMW are shown in Figure 4.

## 3. Results and Discussion

### 3.1. Experimental Results

**Figure 5:**
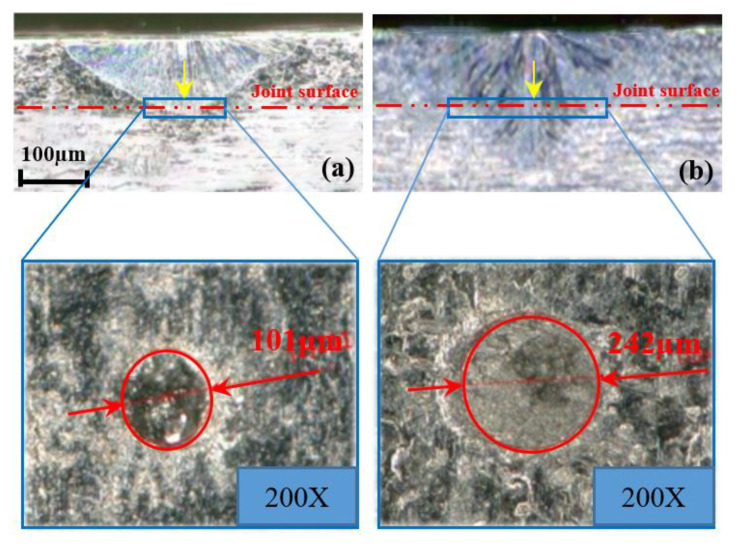
Experimental results of weld morphology and joint surface. (**a**) LEAM, (**b**) LECM.

The metallographic pictures of LSMW welds of LEAM and LECM are shown in Figure 5. As can be seen from the pictures that the weld seam of LEAM is shallow, and the weld depth is only 0.135 mm. On the contrary, LECM has a deeper weld depth up to 0.22 mm. It can be seen from the experimental results that although the total input energy of LEAM (2.0875 J) is higher than the LECM (1.5275 J), the weld depth obtained is different. In LEAM, the depth below the joint surface is only 0.015 mm, and the diameter of the joint surface is just 0.101 mm. It needs to further improve the effective use of laser energy and ameliorate the welding performance in this mode. In contrast, the laser input energy in LECM can be effectively used, and the adhesion between the upper and lower plates is better. However, there will still be slight splash points, and any slight splash is not allowed in the manufacturing process of electronic components, so the waveform of LECM also needs to be further optimized. According to the weld seam depth-to-width ratio (DWR) under the joint surface, the weld seam DWR of LEAM is only 0.148, while the weld seam DWR of LECM can reach 0.413, which is more conducive to the appearance of the “keyhole effect”.

### 3.2. Simulation Results

**Figure 6:**
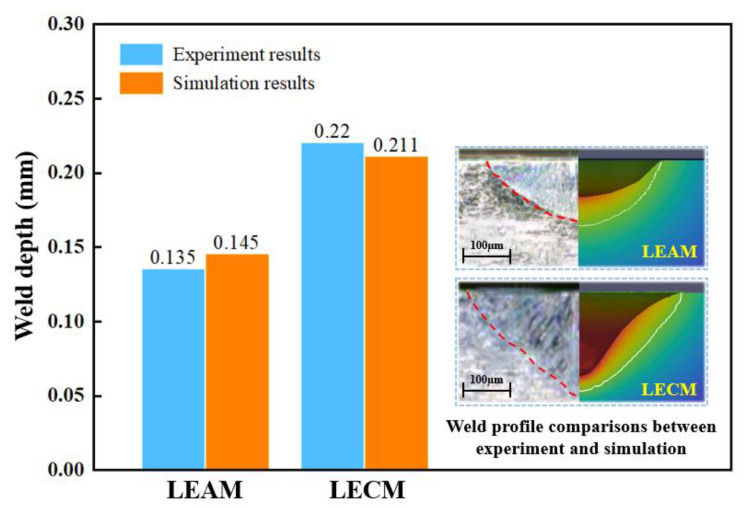
The experimental and simulation results of the weld morphology in LEAM and LECM.

To verify the developed laser spot welding model, the predicted weld dimensions are compared with the experimental results. Figure 6 shows the weld profile comparisons and specific weld depth comparisons. As can be seen from the figure, the simulation results are consistent with the experimental results. The error of the predicted weld depth of both LEAM and LECM is very small, i.e., less than 7%.

**Figure 7:**
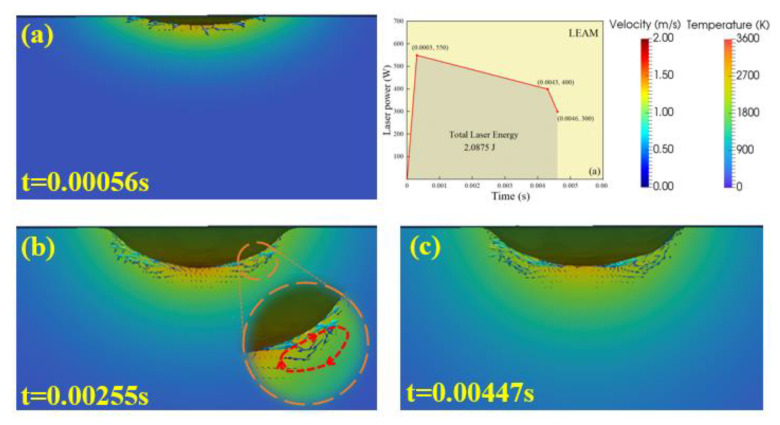
The visual simulation results of the keyhole evolution process in LEAM. (**a**) 0.00056 s, (**b**) 0.00255 s, (**c**) 0.00447 s.

**Figure 8:**
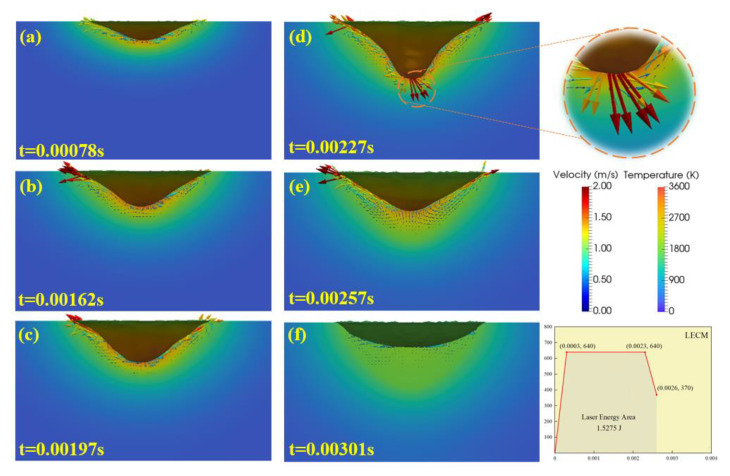
The visual simulation results of the keyhole evolution process in LECM. (**a**) 0.00078 s, (**b**) 0.00162 s, (**c**) 0.00197 s, (**d**) 0.00227 s, (**e**) 0.00257 s, (**f**) 0.00301 s.

Since the entire welding process of the LSMW takes an extremely short time, Figure 7 and Figure 8, respectively, visually analyze the behaviors of the weld pool and the keyhole of LEAM and LECM to understand the influence of different pulse waveforms on the keyhole forming process. In LEAM, a shallow weld pool is formed with uneven temperature distribution, and the highest temperature at the bottom of the keyhole is about 3000 K. Besides, the melting liquid flows upward along the keyhole surface and forms a circulation (Figure 7b), and the maximum magnitude of the surface fluid flow velocity is just about 0.91 m/s. Finally, the metal liquid around the weld pool is backfilled instantly, and the vortex flow gradually weakens (Figure 7c). Throughout the welding process of LEAM, the weld depth is just 0.145 mm. In contrast, the keyhole evolution process changes greatly with time in LECM. The depth of the keyhole is shallow in the early stage (Figure 8a) and then forms the shape with a deep and narrow bottom (Figure 8d). In this mode, the magnitude of the surface fluid flow velocity is about 1.93 m/s, and the weld depth can reach the deepest point of 0.211 mm. This mode can produce an ideal weld depth so that the upper and lower plates are well welded. Besides, at the keyhole center of LECM, the temperature could be up to 3319 K, which is about 300 K higher than the temperature of LEAM.

From the above simulation results, the laser pulse waveforms in LEAM and LECM will significantly affect the forming process of the keyhole. To obtain an ideal solder joint, this article further explores the mechanism of the different waveforms of LEAM and LECM on the LSMW process.

### 3.3. Mechanism of the Difference in Weld Depth under Different Waveforms

The recoil pressure is the main reason for the formation of the keyhole in the laser welding process, and there is a direct interaction between the recoil pressure and the keyhole wall, which determines the stability of the welding process and affects the welding quality [43]. The temperature change inside the keyhole during the welding process changes the recoil pressure [44,45]. Therefore, studying the temperature inside the keyhole of the LSMW process can help to further explore the mechanism of the difference in weld depth under different waveforms.

**Figure 9:**
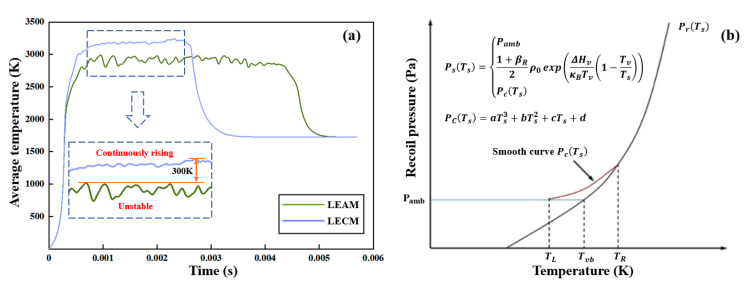
(**a**) Comparison of the average temperature inside the keyhole of LEAM and LECM. (**b**) Diagram of the recoil pressure dependence with temperature.

**Figure 10:**
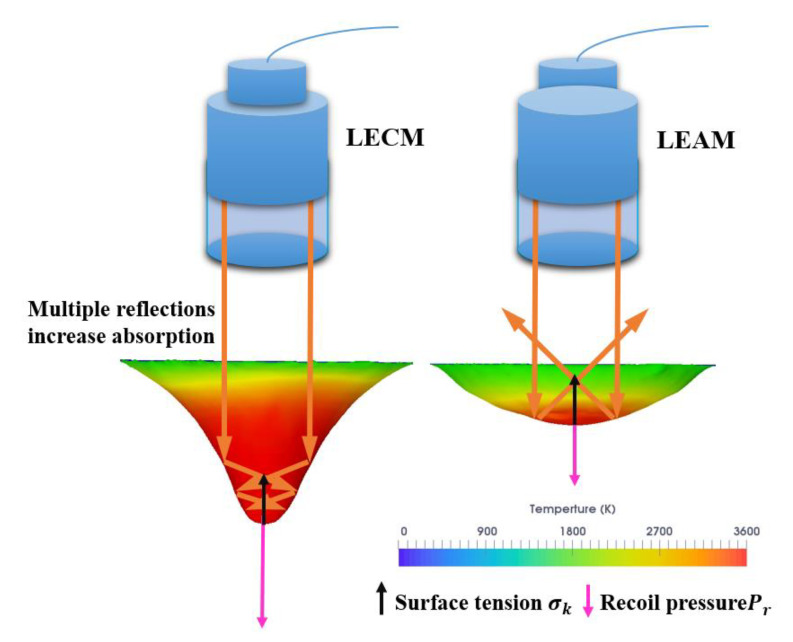
Diagram of temperature distribution and laser reflection path in the keyhole of different modes.

Figure 9a shows the comparison of the average temperature inside the keyhole of LEAM and LECM. The temperature of LECM is higher than that of LEAM, and the temperature inside the keyhole of LECM gradually rises throughout the welding process. When the laser continues to heat for too long, it causes the internal temperature of the melt pool to rise continuously (as shown in Figure 9a). The excessive temperature tends to exceed the boiling point of the base material, and violent evaporation and spattering occur. On the contrary, the temperature inside the keyhole of LEAM fluctuates significantly, and the temperature of the whole welding process shows a downward trend. On the whole, the peak temperature difference in the two modes is about 300 K. This large temperature difference causes the different recoil pressures in LEAM and LECM (Figure 9b) [43,44,45]. As a result, the size of the keyhole and the laser energy distribution in the keyhole are changed. As shown in Figure 10, when the recoil pressure $P_{r}$ increases, it will overcome the effect of surface tension $\delta_{k}$ and produce a larger keyhole depth. In LECM, due to the higher temperature in the keyhole, the keyhole depth under the action of the recoil pressure is larger than that in LEAM. Therefore, multiple reflections of the laser beam are performed on the inner wall of the deeper keyhole wall in LECM, and the laser energy at the bottom of the keyhole is relatively concentrated. On the other hand, the laser beam in LEAM has few reflections in the keyhole wall and even directly reflects into the atmosphere. Eventually, most of the temperature in this mode will be lost. Due to the downward trend of the overall temperature in LEAM, it cannot generate a large recoil pressure, and it is difficult to maintain the large keyhole depth.

According to the above analysis, the larger keyhole depth can help the laser beam to perform multiple reflections inside the keyhole wall and promote the absorption of laser energy by the welding plates. The effective optimization of the temperature in the keyhole is the key factor in increasing the keyhole depth.

### 3.4. Effective Optimization of the Laser Pulse Waveform

To effectively optimize the temperature inside the keyhole in the LSMW process, a temperature improved method combining LEAM and LECM is proposed in this paper. The continuous input energy generated by peak laser power is used to make the base material quickly reach the melting point and get a greater weld depth. Then, the attenuation input energy generated by the attenuation laser power is used to ensure the stability of the temperature during the LSMW process. In the LSMW process, part of the laser energy is reflected and absorbed in the keyhole, and the other part is lost in the air by thermal convection, thermal radiation, and evaporation. Therefore, combined with Equation (4), which uses gaussian distribution as the heat source, Equation (5) lists the heat balance equation in the LSMW process, as shown below:

(5) $$Q=q_{I}+q_{cov}+q_{rad}+q_{v}$$

where $q_{I}$ represents the reflection and absorption of the laser heat in the keyhole wall, while $q_{cov}$, $q_{rad}$,  and $q_{v}$ represent thermal convection, thermal radiation, and evaporative heat dissipation, respectively. The details are shown in Equations (6)–(9) [40,41,42,43,44,45]:

(6) $$q_{I}=\alpha \left(\theta \right)Q+\left[1-\alpha \left(\theta \right)\right]Q\alpha_{mf}\left(\theta \right)$$

(7) $$q_{cov}=h_{c}\left(T_{s}-T_{\infty }\right)$$

(8) $$q_{rad}=k_{B}\varepsilon \left(T_{s}^{4}-T_{\infty }^{4}\right)$$

(9) $$q_{v}=L_{v}\sqrt{m/2\pi k_{B}T_{s}}\cdot P_{s}\left(T_{s}\right)$$

where α(θ) is the first reflection absorption coefficient, $\alpha_{mf}\left(\theta \right)$ is the reflection absorption coefficient for multiple times, $h_{c}$ is the convection heat dissipation coefficient, $T_{\infty }$ is the ambient temperature, $k_{B}$ is the Boltzmann constant, ε is the material emission rate, $L_{v}$ is the latent heat. By switching the terms, we get Equation (10). Finally, a positive correlation between laser power  P(t) and temperature $T_{s}$ is obtained, which is shown in Equation (11).

(10) $$Q=\frac{h_{c}\left(T_{s}-T_{\infty }\right)+k_{B}\varepsilon \left(T_{s}^{4}-T_{\infty }^{4}\right)+L_{v}\sqrt{m/2\pi k_{B}T_{s}}\cdot P_{s}\left(T_{s}\right)}{\left[1-\alpha \left(\theta \right)\right]\cdot \left[1-\alpha_{mf}\left(\theta \right)\right]}$$

(11) $$P\left(t\right)\sim Q\sim f\left(T_{s}\right)$$

Hence, to reasonably use the laser input energy to achieve effective optimization of the temperature in the keyhole during the LSMW process, the laser power is set as a function that can be adjusted over time. For an arbitrarily determined waveform, there will be a certain number of power adjustment points, as well as the time $t_{i}$ and power value $P_{i}$ of each power adjustment point. The laser output power can be written by the following function:

(12) $$P\left(t\right)=P_{i}+\left(P_{i+1}-P_{i}\right)\frac{t-t_{i}}{t_{i+1}-t_{i}},\;\;\;\;\;\;t_{i}<t\leq t+1,\;\;\;\;i=1,\;2,\;3,\ldots \ldots$$

**Figure 11:**
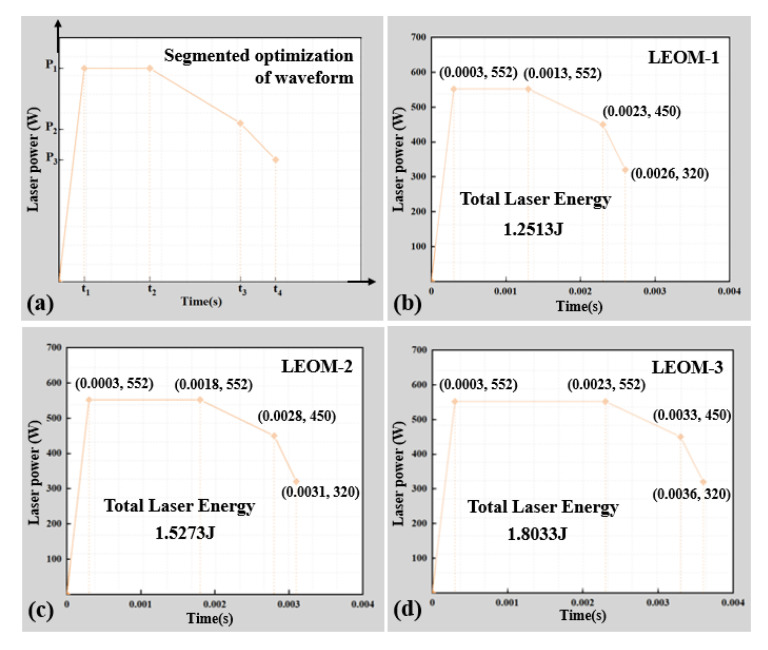
(**a**) A four-segment waveform optimization method. (**b**) LEOM-1; (**c**) LEOM-2; (**d**) LEOM-3. LEOM, laser energy optimization mode.

As shown in Figure 11a, the laser power reaches the peak power *P*_1_ at time *t*_1_ and continues to act until time *t*_2_. During this period, the peak power is continuously inputted to ensure that the plates absorb the laser energy and reach the melting point so that the weld pool bottom quickly reaches through the bonding surface to the lower plate. From time *t*_2_, the laser power begins to decay, and the laser input energy is slowly reduced. This ensures that the temperature of the weld pool is below the material boiling point and further increases the weld depth. From time *t*_3_, the interaction between laser beam and base material gradually weakens, and the whole welding process ends at time *t*_4_. According to the segmented optimization method, the optimized waveforms of laser energy optimization mode (LEOM) are shown in Figure 11b–d. The optimized process parameters are shown in Table 3.

**Figure 12:**
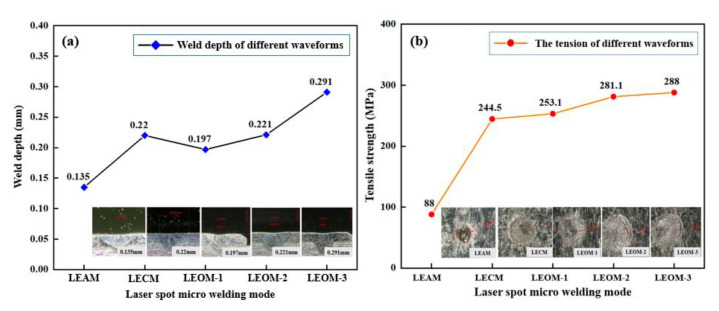
(**a**) Welding depth of different waveforms after and before optimization. (**b**) The tensile strength of different waveforms after and before optimization.

**Figure 13:**
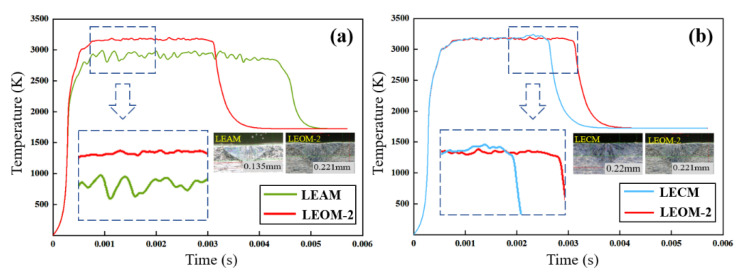
(**a**) Compared with the average temperature of the keyhole center of LEOM-2 and LEAM; (**b**) Compared with the keyhole average temperature of the LEOM-2 and LECM.

The laser spot welding experiments are carried out according to the process parameters set by the optimized waveforms, and the weld depth and the mechanical properties of different modes are obtained, as shown in Figure 12. It can be seen from the figure that the weld depth of the segmented optimization waveforms is on the rise, up to 0.291 mm. The tensile strength has been improved, up to 288 MPa. Compared to before optimization, the solder joint performance has been significantly improved. Figure 13 shows the average temperature in the keyhole center of LEOM-2 compared with both LEAM and LECM. As can be seen from Figure 13a, the average temperature of LEOM-2 is higher than LEAM, and the change in the temperature is more stable. There is no obvious fluctuation phenomenon. Figure 13b shows that the average temperatures of LEOM-2 and LECM are similar. But the temperature of LEOM-2 at the peak power stage is more stable than LECM. This is because LEOM-2 sets the energy attenuation process after the peak power stage, which avoids the continuous rise of the internal temperature of the keyhole, making the whole welding process more stable. It can be seen from the optimized experimental results that the weld depth of LEOM-2 is 0.221 mm, and it is an ideal depth in the LSMW process. As a result, a greater weld depth, bonding ability, and higher welding quality are obtained.

In summary, the optimized four-segment waveform can make the base material reach the melting point in a short time. The optimized waveform not only overcomes the temperature loss in LEAM due to energy attenuation but also makes the welding process generate enough recoil pressure to maintain the opening of the keyhole, better absorb the energy, produce a larger depth of fusion, and obtain the ideal welding spot. It also overcomes the sudden rise in energy at the end of the welding of LECM, keeps the temperature inside the keyhole in a relatively stable process, and avoids spatter when the temperature exceeds the boiling point. The results can help provide effective process guidance for optimization of the laser pulse waveform in the practical engineering application.

## 4. Conclusions

The technology of using pulsed laser for the micro-welding process has the characteristics of fast welding speed and controllable waveform, which is suitable for the connection between ultra-thin metal materials. In this paper, the effect of different laser pulse waveforms in the LSMW process on 304 stainless steel ultra-thin plates is mainly investigated. The major findings are shown as follows:

(1) This paper explores the micro-welding process of the LSMW through experimental and numerical simulation methods. The simulation results show that LECM is a keyhole welding mode, and LEAM tends to be a thermally conductive welding mode. The experimental results show that LECM with low laser input energy can obtain a greater weld depth than LEAM. The weld depth of LECM mode can reach 0.22 mm, while the weld depth of LEAM is 0.135 mm. The tensile strength of LECM mode can reach 244.5 MPa, which is much greater than that of LEAM mode at 88 MPa. Both experimental and simulation results show that LECM mode has better welding performance than LEAM.
(2) Analysis based on the simulation results reveals that the premature attenuation of the laser power in the LSMW process will cause the temperature fluctuation and decline in the keyhole, and it is difficult to generate a large recoil pressure to maintain the opening of the keyhole. Finally, the whole process is more active in the thermal conductivity mode, and a weak weld phenomenon has appeared. On the other hand, because of the peak power, the internal temperature of the keyhole can be at a higher level, resulting in the keyhole effect. It is more conducive to the multiple reflections of the laser beam in the keyhole. Therefore, the LECM mode with less laser energy is able to produce a greater weld depth than LEAM due to the presence of peak power.
(3) A laser pulse waveform segmented optimization method, in this paper, is proposed for the practical engineering application, which combines LEAM and LECM into a four-segment waveform. The laser energy after waveform optimization is well utilized, and the bonding ability is increased. The experimental results show that the weld depth can be optimized to 0.291 mm, and the tensile strength can reach 288 MPa. It can reach the required weld depth in a short time and improve the welding efficiency of the LSMW process. Besides, the simulation results show that the temperature in the keyhole is also well optimized below the material boiling point. It is always in a stable state, and the possibility of welding spatter is reduced to a greater extent.

## Figures and Tables

**Table 1:** Chemical composition of 304 stainless steel sheet (mass fraction, %).

C	Si	Mn	P	S	Ni	Cr	Fe
0.07	0.46	0.78	0.032	0.006	8.10	18.32	Balance

**Table 2:** Experimental process parameters of LSMW on the ANSI 304 stainless steel sheet.

Process Name	LEAM	LECM
Waveform duration (s)	t_1_ = 0.0003, t_2_ = 0.0043, t_3_ = 0.0046	t_1_ = 0.0003, t_2_ = 0.0023, t_3_ = 0.0026
Action laser power (W)	P_1_ = 550, P_2_ = 400, P_3_ = 300	P_1_ = 640, P_2_ = 640, P_3_ = 370
Total input energy (J)	2.0875 J	1.5275 J

**Table 3:** Optimized process parameters of LSMW on the ANSI 304 stainless steel sheet.

Process Name	LEOM-1	LEOM-2	LEOM-3
Waveform duration (s)	t_1_ = 0.0003, t_2_ = 0.0013, t_3_ = 0.0023, t_4_ = 0.0026	t_1_ = 0.0003, t_2_ = 0.0018, t_3_ = 0.0028, t_4_ = 0.0031	t_1_ = 0.0003, t_2_ = 0.0023, t_3_ = 0.0033, t_4_ = 0.0036
Action laser power (W)	P_1_ = 552, P_2_ = 552, P_3_ = 450, P_4_ = 320		
Total input energy (J)	1.2513	1.5273	1.8033

## Appendix A

In this paper, recoil pressure, surface tension, and Marangoni force are properly considered. The level-set method is used to track the weld pool surface, and the ray tracing method is used to treat laser heating. For simplicity, the metal vapor and chemical reactions are not considered, and the weld pool is assumed to be incompressible laminar flow in this model.

### Appendix A.1. Governing Equations

In the process of LSMW, the heat transfer behaviors and mass vary behaviors of the weld pool can be described by three governing equations. Firstly, assuming that the density of 304 stainless steel does not change significantly with temperature [36,37,38,39,40,41,42,43] and considering the solid-to-liquid interaction force inside the weld pool, the mass and momentum conservation equation of the fluid flow of the weld pool can be written as follows:

(A1) $$\nabla \cdot \vec{U}=0$$

(A2) $$\rho (\frac{\partial \vec{U}}{\partial t}+\left(\vec{U}\cdot \nabla \right)\vec{U})=\nabla \cdot \left(\mu \nabla \vec{U}\right)-\nabla p-\frac{\mu }{K}\vec{U}-\frac{C\rho }{\sqrt{K}}\left|\vec{U}\right|\vec{U}+\rho \vec{g}\beta \left(T-T_{ref}\right)$$

where $\vec{U}$ means the three-dimensional velocity, p  means the pressure. ρ, μ, β mean the density, kinematic viscosity of the fluid, and expansion coefficient of the thermal, respectively. $T_{ref}\;$ means the reference temperature. K  means the Carman–Kozeny constant in the mixture-fluid phase model, and C means an inertial coefficient closely related to a liquid fraction.

Considering the convection and conduction of heat inside the weld pool and the heat transfer of the base metal, the energy equation can be described as:

(A3) $$\rho C_{p}\left(\frac{\partial T}{\partial t}+\left(\vec{U}\cdot \nabla \right)T\right)=\nabla \cdot \left(k\nabla T\right)$$

where $\;C_{p}$ stands for the specific heat capacity, k stands for the thermal conductivity of the metal fluid, and T stands for the temperature.

In this study, the level-set method is used to track the evolution process of the weld pool free interface, and the equation can be described as [40]:

(A4) $$\frac{\partial \emptyset }{\partial t}+\vec{U_{k}}\cdot \nabla \emptyset =0$$

where ∅  represents the signed distance field function. The weld pool free surface is represented by  ∅ = 0, ∅ < 0 represents the metal area, and  ∅ > 0  is the gas area.

The curvature value k and the normal vector $\vec{n}$ at any point on the free interface can be written as follow:

(A5) $$k=\nabla \cdot \frac{\nabla \emptyset }{\left|\nabla \emptyset \right|}$$

(A6) $$\vec{n}=\frac{\nabla \emptyset }{\left|\nabla \emptyset \right|}$$

### Appendix A.2. Boundary Conditions

Based on previous work [36,37,38,39,40,41,42,43], these above-mentioned forces can be loaded with the sharp-interface method. In this study, the conditions of momentum boundary on the surface of the weld pool can be written as:

(A7) $$P_{f}=P_{r}+\delta k+2\mu_{l}\vec{n}\cdot \vec{U_{l}}\cdot \vec{n}$$

(A8) $$\begin{array}{c} (\mu_{l}\nabla \vec{U_{l}})=\mu_{l}\left(\vec{n}\;{\vec{t}}_{1}\;{\vec{t}}_{2}\right){\left(\vec{n}\;\vec{0}\;\vec{0}\right)}^{T}\left(\nabla \vec{U_{l}}\right)\left(\vec{n}\;\vec{0}\;\vec{0}\right){\left(\vec{n}\;{\vec{t}}_{1}\;{\vec{t}}_{2}\right)}^{T}+\mu_{l}\left(\vec{n}\;{\vec{t}}_{1}\;{\vec{t}}_{2}\right){\left(\vec{0}\;{\vec{t}}_{1}\;{\vec{t}}_{2}\right)}^{T}\left(\nabla \vec{U_{l}}\right)\\ \;-\mu_{l}\left(\vec{n}\;{\vec{t}}_{1}\;{\vec{t}}_{2}\right){\left(\vec{n}\;\vec{0}\;\vec{0}\right)}^{T}\left(\nabla \vec{U_{l}}\right)\left(\vec{0}\;{\vec{t}}_{1}\;{\vec{t}}_{2}\right){\left(\vec{n}\;{\vec{t}}_{1}\;{\vec{t}}_{2}\right)}^{T}\\ +\left(\vec{n}\;{\vec{t}}_{1}\;{\vec{t}}_{2}\right)\left[\begin{array}{ccc} 0 & \nabla_{s}\sigma \cdot {\vec{t}}_{1} & \nabla_{s}\sigma \cdot {\vec{t}}_{2}\\ 0 & 0 & 0\\ 0 & 0 & 0 \end{array}\right]{\left(\vec{n}\;{\vec{t}}_{1}\;{\vec{t}}_{2}\right)}^{T} \end{array}$$

where  δ means the surface tension coefficient, $P_{f}$ means the free interface pressure, $P_{r}\;$ means the recoil pressure, $\;{\vec{t}}_{1}$ and $\;{\vec{t}}_{2}$ are the orthogonal tangent vectors perpendicular to $\vec{n}$. $\nabla_{s}$ means the surface gradient operator, which can be expressed as $\nabla_{s}=\left(I-\vec{n}\vec{n}\right)\nabla$, and $\nabla_{s}\sigma$ is the Marangoni force.

Assuming that the energy of the laser beam is Gaussian distribution, the distribution function of laser beam energy at time t and the laser energy density Q absorbed by the liquid surface micro-element at time t can be written as:

(A9) $$I\left(t,r,z\right)=\frac{3Q}{\pi R^{2}}exp\left(-\frac{3r^{2}}{R^{2}}\right)$$

(A10) $$Q=I_{0}\left(r,z\right)\left({\vec{I}}_{0}\cdot {\vec{n}}_{0}\right)\alpha_{F_{r}}\left(\theta_{0}\right)+\overset{N}{\underset{m=1}{\sum }}I_{m}\left(r,z\right)\left({\vec{I}}_{m}\cdot {\vec{n}}_{m}\right)\alpha_{F_{r}}\left(\theta_{m}\right)$$

where R represents the laser spot radius, Q represents the laser energy density. $\alpha_{F_{r}}\left(\theta_{0}\right)$ represents the Fresnel absorption coefficient, N represents the total incidence of multiple reflections under consideration, generally no more than 3, θ represents the angle between the laser and the keyhole wall normal vector. $I_{0}\left(r,z\right)$ represents the laser energy distribution. $\vec{I}$ and $\vec{n,}$ respectively, represent the laser beam direction and the normal vector of the keyhole wall after the normalization method [36,37,38,39,40,41,42,43].

Technically speaking, the precise discretization of the equations and the development of effective solvers are very important for the simulation of LSMW. In this paper, the flow equations of the weld pool (including Equation (A1) mass conservation and Equation (A2) momentum conservation equation) are calculated by the efficient projection method, and the diffusion terms of Equations (A4) and (A5) are discretely solved using the center difference format. In Equation (A5), the left term is the energy time term and the convection heat transfer term. In the solution of the convection term, the Weighted Essentially Non-Oscillatory (WENO) format is used for discretization. While in Equation (A3), the semi-implicit scheme is used for the discretization of the energy equation.

### Appendix A.3. Simulation Parameters

Numerical simulations of LSMW are performed by using the above-mentioned model. The same process parameters as the experiments are used in the simulations. To reduce the cost of calculation, the computational domains in this paper are set to 1.4 mm × 1.4 mm × 0.42 mm. A uniform grid size of 0.01 mm is used. The physical parameters used are shown in Table A1 [44,45].

**Table A1:** Physical parameters in the LSMW simulation process.

Physical Parameters	Units	Value
Density ρ	$\mathrm{kg}/m^{3}$	7200
Thermal conductivity coefficient k	W/m·K	35 (298 K)
Specific heat $C_{p}$	J/kg·K	760 (298 K)
Kinematic viscosity μ	Kg/m·s	0.006
Latent heat of fusion $C_{f}$	J/kg	6 × 10^5^
Latent heat of evaporation $C_{v}$	J/kg	6.52 × 10^6^
Solidus temperature $T_{s}$	K	1727
Liquidus temperature $T_{l}$	K	1697
Evaporation temperature $T_{v}$	K	3100
Surface tension coefficient σ	N/m	1
Thermal-capillary force coefficient $\sigma_{T}$	N/m·K	−0.43 × 10^−3^
